# Case report: Multi-detector CT of left pulmonary artery sling

**DOI:** 10.4103/0971-3026.40292

**Published:** 2008-05

**Authors:** Charan Kamal Singh, Sanjiv Sharma, SS Kothari, Priya Jagia

**Affiliations:** Department of Cardiac Radiology, All India Institute of Medical Sciences, Ansari Nagar, New Delhi - 110 029, India; 1 Department of Cardiology, All India Institute of Medical Sciences, Ansari Nagar, New Delhi - 110 029, India

Left pulmonary artery sling (LPAS) is a rare vascular anomaly in which the left pulmonary artery (LPA) arises from the right pulmonary artery (RPA) and enters the left hilum after passing between the trachea and the esophagus; it can cause respiratory distress. This case demonstrates the role of multi-detector CT (MDCT) in the diagnosis of LPAS and associated cardiovascular and tracheobronchial anomalies.

## Case Report

**Figure 1(A-C):**
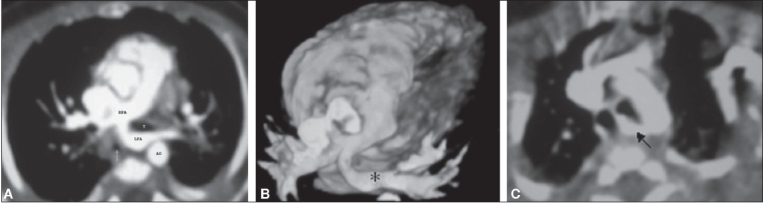
MDCT. Axial volumetric reconstruction (A) shows the LPA arising from the RPA, consistent with a pulmonary artery sling. The LPA is seen coursing between the trachea (T) and esophagus (arrow). The volume-rendered image (B) shows the characteristic course of the aberrant LPA (FNx01). An associated aberrant right subclavian artery (arrow) is also seen more superiorly (C)

A 4-month-old male infant with Down's syndrome and atrial septal defect presented with respiratory distress and recurrent pneumonia. A chest radiograph revealed reduced aeration of the left lung as compared to the right. The echocardiogram showed poor visualization of the pulmonary confluence and the proximal LPA. CT scan was performed on a 16-slice CT scanner (Sensation-16, Siemens, Germany). Iodinated non-ionic contrast, 2 ml/kg body weight, was injected using a pressure injector. Axial source and volume-rendered images revealed the LPA arising from the proximal RPA and coursing to the left hilum, between the trachea and the esophagus [Figure 1A, B]. Mildly reduced lung volume was also noted on the left. An associated aberrant right subclavian artery, coursing behind the esophagus was also detected [Figure 1C].

**Figure 2:**
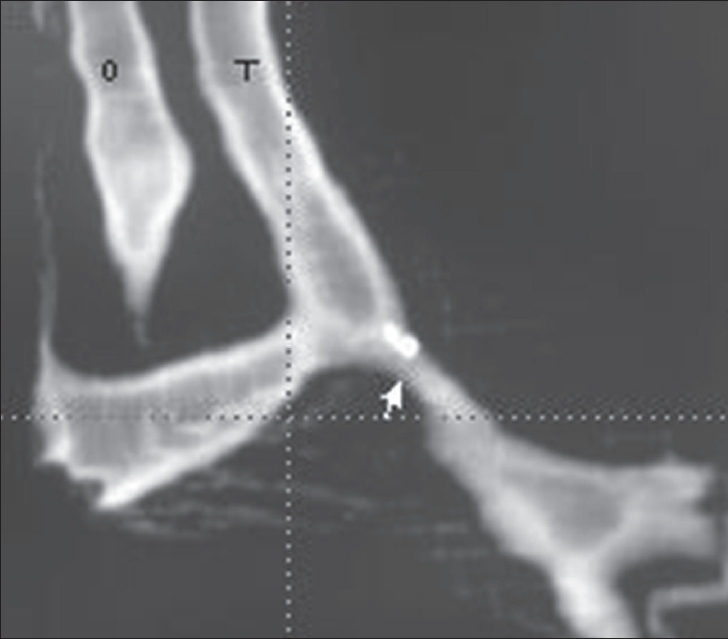
Three-dimensional image of the tracheobronchial tree shows stenosis of the proximal left main bronchus (arrow) and vascular indentations on the trachea (T) and esophagus (O)

A volume-rendered image, obtained for the tracheobronchial anatomy [Figure 2], revealed long-segment stenosis of the left main bronchus, besides showing the vascular indentations by the LPA on the trachea and the esophagus, as well as the impression of the aberrant subclavian artery on the esophagus.

The child was operated upon successfully, with reimplantation of the LPA into the main pulmonary artery and bronchoplasty.

## Discussion

The first case of pulmonary artery sling was reported by Glaevecke and Doehle in 1897.[1] Embryologically, it is thought to be due to involution of the proximal left sixth arch.[2] The anomalous LPA is formed by a collateral vessel from the RPA in response to the lack of arterial supply to the left side, thus linking the primitive circulations.[3] In the area where this anomalous LPA passes behind the bronchus there is likely to be bronchial compression, resulting in functional stenosis. Chen *et al*.[4] believe that tracheal stenosis and LPAS are both primary conditions that occur together as part of the developmental anomaly. They also espouse the “space availability concept” wherein the development of the associated patent ductus arteriosus and persistent left superior vena cava are due to the increased space on the left, where the normal LPA would otherwise have been placed.

Fifty percent of affected infants are symptomatic at birth, while by one month of age, two-thirds of infants have clinical symptoms.[5] The commonest clinical presentations include recurrent respiratory distress, stridor, and wheezing. The mortality rate is up to 90% in medically managed cases.[6] Even after surgical correction, LPAS can be fatal in up to 50% of cases.[5]

The prognosis is affected by the presence of associated tracheobronchial tree anomalies, especially a long tracheobronchial stenosis.[7] The tracheobronchial anomalies may include a bronchus arising directly from the trachea to supply a segment of the right upper lobe (bronchus suis), hypoplasia of the distal trachea, incomplete cartilaginous tracheal ring, and left main bronchial stenosis.[3] Cardiovascular anomalies consist of atrial septal defect, patent ductus arteriosus, ventricular septal defect with pulmonary stenosis, tetralogy of Fallot, aortic arch anomalies, and persistent left superior vena cava.[4,5] Therefore, optimal preoperative imaging of the tracheobronchial and cardiovascular anatomy is essential for planning surgical management.[8–10]

MDCT helps avoid invasive investigations such as bronchoscopy and pulmonary artery angiography.[11] The associated cardiovascular and tracheobronchial anomalies too, are easily detected. In this case, CT images were considered adequate for the diagnosis and assessment of the anatomical relationship of the tracheobronchial tree, prior to surgical management.
